# An analysis of compensation for radial thermal errors of a turning center with a three axis feed system

**DOI:** 10.1038/s41598-025-05804-5

**Published:** 2025-07-02

**Authors:** Zhao Haitao, Guo Jingfeng, Tang Yongbo, Zhang Shuixiang, Yi Xiaoxi

**Affiliations:** Physical Science and Technology College of Yichun University, Yichun, 336000 China

**Keywords:** Turning center, Three-axis feed system, Radial thermal error, Compensation, Spindle axis, Mechanical engineering, Applied mathematics

## Abstract

Under normal cutting conditions, the cutting tool tip passes through the spindle axis when it moves along X axis during the whole turning session, however, which is difficult to realize for the turning centers with the conventional two feed axes due to the spindle axis movement caused by the thermal deformation of machine tool structures. Addressing this problem, a novel three-axis feed system is proposed in this paper. The effect of the thermal movement of the spindle axis on the radial thermal error is analyzed. The linear and angular components of radial thermal error are derived from the proposed three-axis feed system. The whole compensation process is demonstrated using a turning center equipped with this feed system, and the needed data is mainly from simulation. The effects of the parameter *l* of this feed system on the two radial thermal error components are analyzed. Furthermore, the three-axis feed system can also be used as a two-axis feed system by locking the revolving feed axis, which can make the cutting tool tip pass through the spindle axis easier than the conventional two-axis feed systems, and therefore it is helpful in both facilitating the adjusting or assembling process of the turning center and reducing radial thermal errors of machined parts.

## Introduction

The turning center is widely used to machine rotational parts, and its machining accuracy is affected by many factors among which the thermally induced errors account for a larger part^1^. Many methods and technologies have been developed to control thermal errors during the past years. Lingtao Weng et al.^2^ proposed an analytical modelling method for thermal balancing design of machine tool structural components, which can be used to optimize the thermal layout of machine tool components so as to reduce thermal deformation magnitude. Teng Liu et al.^3^ developed a differentiated multi-loops bath recirculation system which can accomplish differentiated and close-loop cooling strategies onto machine heat generating parts during its operation and thus beneficial for heat dissipation. According to Makoto Fujishima^4^, based on the results from the sensitivity analysis, geometry of thermally sensitive section of the machine structure was improved to build more thermally robust machine. The above technologies are mainly developed from the angle of prevention, which can only be better applied to the design period of machine tool, not appropriate for the existing machine tool, and therefore more and more works are simultaneously focused on compensation for the thermal errors.

With regard to compensation for thermal errors, the first step is to build the thermal error model that matches the feeding system. When compensation, the thermal errors are predicted in real time by the thermal error model and decomposed to the related feed axes of the feeding system to realize the thermal error compensation. The predicting accuracy of thermal errors determine the compensation accuracy, so the thermal error modeling technologies and related temperature measuring points selection technologies have become the major issues. The traditional linear regression models still play an important role in predicting thermal errors up to now. Chethana R. Gowda et al.^5^ used multiple linear regression model on a two-axis turning center and analyzed the relations between the number of data and model accuracy, while Nabil Ouerhani et al.^6^ compared the performances of four Machine Learning algorithms that were used to predict thermal errors on a turning machine tool, and found the classical linear regression models were still able to predict thermal errors with high accuracy. With the development of artificial intelligence, more and more thermal error models^7–9^ have the characteristics of self-improvement. Nico Zimmermann et al.^10^ proposed a method which combines the Group-LASSO (least absolute shrinkage and selection operator) for autoregressive models with exogenous inputs (ARX) and the particle swarm optimization to realize a simultaneous estimation of the optimal inputs, the model structure, and the model parameters. Furthermore, the self-optimization ability of thermal error models, based on the TALC, is increased by introducing error-specific action control limits to define the frequency of model updates. In^11^, a novel use of a Kalman filter together with model order reduced finite element models is presented to observe the entire thermal state, which allows the subsequent solution of the mechanical model and computation of the thermal errors in real-time without requiring any training data but instead purely based on the physical system model. Due to the limit of the number of temperature sensors on the machine tool structure, selection of the positions of temperature sensors will affect the prediction accuracy of thermal error models, so the related works have been done simultaneously with thermal error modeling^12^. Guolong Li et al.^13^ proposed a new temperature-sensitive point screening method based on the Improved Binary Grasshopper Optimization Algorithm (IBGOA) feature selection. In^14^, a transfer function matrix and a sensitivity function were defined to optimize the number and locations of sensors without physically attaching them to the machine tool. With the progress of data-driven manufacturing, it has become a trend to prioritize the use of manufacturing data for achieving precise analysis and control, rather than relying solely on simplified physical models and human expertise, and the related issues are discussed in^15^.

**Fig. 1:**
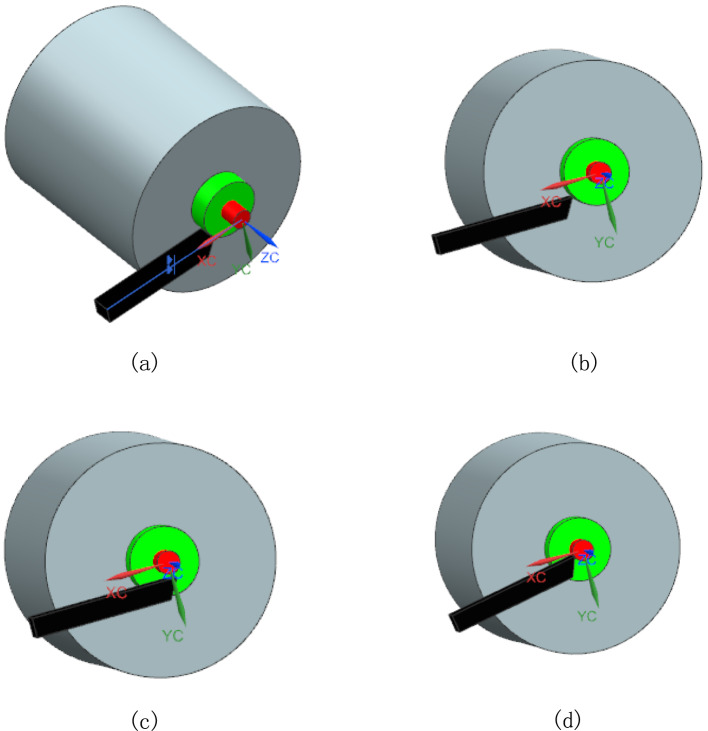
The cutting status with and without rotational feed axis (Created using NX12.0, it is available at the URL: https://plm.sw.siemens.com/zh-CN/nx/).

It is notable that the feeding systems of the turning centers used in the above study works only have two feeding axes, that is, X-axis and Z-axis, and the radial error can only be compensated by the X-axis feeding movement, which inevitably results in such problems as stated in^16,17^ since the thermal deformation can lead to changes in the position of the spindle axis and thus make the X-axis not intersect with the spindle axis. Extremely, when the radial dimensions of the machined part approach the magnitude of the change of spindle axis position, it will be in vain to compensate the radial thermal error by the X-axis feeding movement. All these situations can be more clearly seen from Fig. 1. In Fig. 1, the coordinate system shown is the machining coordinate system, whose three axial directions are fixed and the feeding X-axis of the cutter is along the cutter length. Figure 1a represents the initial status where no thermal movement of the spindle axis occurs and the feeding X-axis of the cutter is parallel to the X axis of the machining coordinate system and pass through the spindle axis. Figure 1b and c depict the effect of thermal movement on the spindle axis using a conventional 2-axis feed system. The feeding X-axis of the cutter remains parallel to the X-axis of the machining coordinate system but does not pass through the spindle axis. Consequently, the radial thermal error of the green cylinder surface can be compensated, whereas that of the red cylinder surface cannot because of its smaller radial dimension. In order to compensate for the radial thermal error of the red cylinder surface, the feeding X-axis of the cutter needs to be rotated at an angle as shown in Fig. 1d. To address these issues, a novel three-axis feed system is presented in this paper, and the detailed process concerning the compensation for radial thermal errors with this feed system is analyzed. At last, an application example on a turning center is demonstrated.

## The radial thermal error caused by movement of the spindle axis

Due to the slower feeding velocity and smaller heat generation, the feeding system usually leads to a relative smaller thermal deformation, and therefore the thermally induced movement of the spindle axis is the major cause for thermal errors. The spindle is fitted with bearings into the spindle hole in the headstock, and therefore the spindle axis always remains aligned with the spindle hole axis which can be determined by the positions of several points on the hole wall. The revolving bearings at high speed are the main thermal sources that make the headstock temperature rise and thus produce thermal deformation. In most cases the headstock structures on both ends are thermally symmetrically designed, and thus the deviation angle of the spindle axis is very small and neglectable, which has been verified by the simulation results in section “Demonstration and analysis of compensation process”, so it is assumed here that the spindle axis only takes translational movement and the spindle hole keeps the form of a circle during the whole thermal deformation process.

**Fig. 2:**
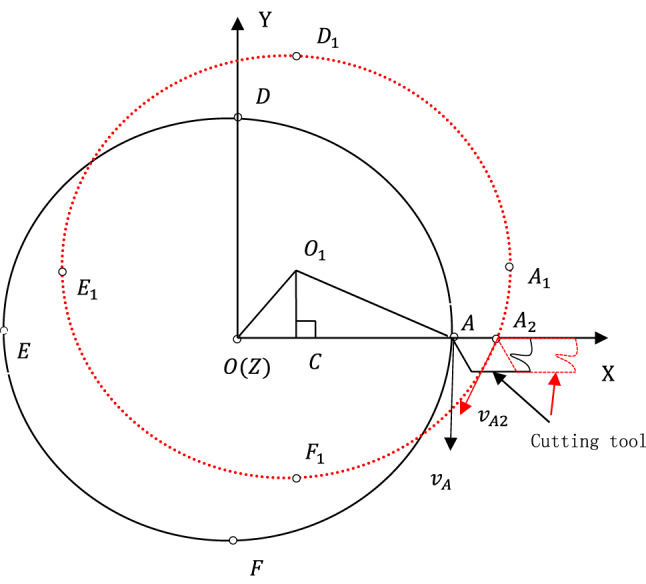
The thermal movement of the spindle axis.

Based on the above assumption, the thermal movement of the spindle axis can be shown clearly on the Z-direction view, as illustrated in Fig. 2. The black solid circle and the red dotted circle represent the positions of the spindle hole (also the spindle or the workpiece since they are always coaxial) before and after thermal deformations respectively, and their center O and O_1_ represent the position of the spindle axis before and after thermal deformations respectively. Points E, A, F and D represent the left, right, lower and upper extreme point of the spindle hole before thermal deformation respectively, while points E_1_, A_1_, F_1_, and D_1_ represent the left, right, lower and upper extreme point of the spindle hole after thermal deformation respectively. The coordinates (${O}_{x}$, ${O}_{y}$) of point O can be expressed as:

1 $${O}_{x}={E}_{x}+\frac{1}{2}\left({A}_{x}-{E}_{x}\right)$$

2 $${O}_{y}={F}_{y}+\frac{1}{2}\left({D}_{y}-{F}_{y}\right)$$

where subscript x and y represent the x coordinate and y coordinate of points respectively. Similarly, the coordinates (${O}_{\text{1x}}$, ${O}_{\text{1y}}$) of point O_1_ can be expressed as:

3 $${O}_{\text{1x}}={E}_{1x}+\frac{1}{2}\left({A}_{1x}-{E}_{1x}\right)$$

4 $${O}_{\text{1y}}={F}_{\text{1y}}+\frac{1}{2}\left({D}_{\text{1y}}-{F}_{\text{1y}}\right)$$

The movement of the spindle axis can be shown by the vector $$\overset{\lower0.5em\hbox{$\smash{\scriptscriptstyle\rightharpoonup}$}}{{OO_{1} }}$$ that can be written as:

$$\overset{\lower0.5em\hbox{$\smash{\scriptscriptstyle\rightharpoonup}$}}{{OO_{1} }} = \left[ {E_{{1x}} - } \right.E_{x} + \frac{1}{2}\left( {A_{{1x}} - E_{{1x}} } \right) - \frac{1}{2}\left( {A_{x} - E_{x} } \right),~~~~\left. {F_{{{\text{1y}}}} - F_{y} + \frac{1}{2}\left( {D_{{{\text{1y}}}} - F_{{{\text{1y}}}} } \right) - \frac{1}{2}\left( {D_{y} - F_{y} } \right)} \right]$$

It can also be rewritten as the addition of the following two vectors:

$$\overset{\lower0.5em\hbox{$\smash{\scriptscriptstyle\rightharpoonup}$}}{{OO_{1} }} = \left[ {E_{{1x}} - } \right.E_{x} ,~~~~\left. {F_{{{\text{1y}}}} - F_{y} } \right] + \left[ {\frac{1}{2}\left( {A_{{1x}} - E_{{1x}} } \right) - \frac{1}{2}\left( {A_{x} - E_{x} } \right),~\frac{1}{2}\left( {D_{{{\text{1y}}}} - F_{{{\text{1y}}}} } \right) - \frac{1}{2}\left( {D_{y} - F_{y} } \right)} \right]$$

where the first vector $\left[{E}_{1x}-\right.{E}_{x}, \left.{F}_{\text{1y}}-{F}_{y}\right]$ can be regarded as the spindle axis movement caused by the thermal deformation of the headstock base when the dimensions of the spindle hole don’t change, and the second vector $\left[\frac{1}{2}\left({A}_{1x}-{E}_{1x}\right)-\frac{1}{2}\left({A}_{x}-{E}_{x}\right), \frac{1}{2}\left({D}_{\text{1y}}-{F}_{\text{1y}}\right)-\frac{1}{2}\left({D}_{y}-{F}_{y}\right)\right]$ can be regarded as the spindle axis movement caused by the enlargement of the spindle hole.

It can be seen from Fig. 1 that the radial thermal error ${E}_{radial}$ can be expressed as:

5 $${E}_{radial}=OA-{O}_{1}A=OA-\sqrt{{\left(OA-\left({O}_{1x}-{O}_{x}\right)\right)}^{2}+{({O}_{1y}-{O}_{y})}^{2}}$$

where $OA$ and ${O}_{1}A$ can be seen as the ideal and practical radial dimensions respectively, so the radial thermal error is partly determined by the radial dimension $OA$ itself. The derivative of (5) with regard to $OA$ is

6 $$\frac{{\partial E_{radial} }}{\partial OA} = \left\{ {\begin{array}{*{20}l} {1 - \frac{1}{{\sqrt {1 + \left( {\frac{{O_{1y} - O_{y} }}{{OA - \left( {O_{1x} - O_{x} } \right)}}} \right)^{2} } }}, { }} \hfill & {{\text{if}}\quad OA - \left( {O_{1x} - O_{x} } \right) \succ 0} \hfill \\ {1,{ }} \hfill & {{\text{if}}\quad OA - \left( {O_{1x} - O_{x} } \right) = 0} \hfill \\ {1 + \frac{1}{{\sqrt {1 + \left( {\frac{{O_{1y} - O_{y} }}{{OA - \left( {O_{1x} - O_{x} } \right)}}} \right)^{2} } }},{ }} \hfill & {{\text{if }}\quad OA - \left( {O_{1x} - O_{x} } \right) \prec 0} \hfill \\ \end{array} } \right.$$

The above formula shows the velocity of change of the radial thermal error with the change of the radial dimension of the to-be-machined workpiece. Usually $OA$ is significantly larger than both ${O}_{1x}-{O}_{x}$ and ${O}_{1y}-{O}_{y}$, because the magnitude of thermal deformation is small. Therefore, when $OA$ is larger, the partial derivative of radial thermal error with respect to $OA$, $\frac{\partial {E}_{radial}}{\partial OA}$, approximates 0, resulting in the radial thermal error being almost constant. The constant can be obtained by solving the limit of formula (5):

7 $$\underset{OA\to \infty }{lim}{E}_{radial}=\underset{OA\to \infty }{lim}\left(OA-\sqrt{{\left(OA-\left({O}_{1x}-{O}_{x}\right)\right)}^{2}+{({O}_{1y}-{O}_{y})}^{2}}\right)={O}_{1x}-{O}_{x}$$

The formula (7) shows that the radial thermal error is completely determined by the thermal displacement of the spindle axis in the X-axis direction when the radial dimension is large enough, and therefore it can be compensated by the feeding movement of X-axis. It can be seen from Fig. 2 that, when moving the cutting tool tip from A to A2 (from the black position to the red position of the cutting tool), the radial thermal error will be perfectly compensated; nonetheless, the cutting speed direction undergoes significant changes from ${v}_{A}$ at point A to ${v}_{A2}$ at point A_2_, which can result in the poor cutting conditions and consequently, poor surface finish.

On the other hand, when the radial dimension $OA$ is smaller, there will be a larger $\frac{\partial {E}_{radial}}{\partial OA}$ value which means the radial thermal error changes faster with the changes of $OA$; furthermore, the thermal displacement ${O}_{1y}-{O}_{y}$ of the spindle axis in the Y-axis direction can’t be neglected. It can be seen from Fig. 1 that the radial thermal error cannot be compensated only by the feeding movement of X-axis when the radial dimension is smaller than ${O}_{1}C$. Though such small radial dimension is rare, it is necessary to find a solution to eliminate the potential degradation of machining accuracy.

Based on the above analysis, the thermal deformation in the headstock causes movement of the spindle axis, resulting in a non-intersection between the spindle axis and the feeding X-axis. This non-intersection ultimately means that the radial thermal error cannot be perfectly compensated solely by the feeding movement of the X-axis. To address this problem, this paper introduces a novel three-axis feed system capable of rotating the X-axis component, ensuring that the spindle axis always intersects with the X-axis throughout the entire machining process. The subsequent sections will delve into the specific structure of this novel feed system and provide detailed analysis of its applications.

## The three-axis feed system for a turning center

Under normal conditions, a turning center only has two feed axes: X-axis that controls the radial dimensions and Z-axis that controls axial dimensions of a revolving workpiece. However, as demonstrated in the above analysis, the radial thermal error can’t be compensated perfectly only by the feed movement of X-axis due to the thermal displacement of the spindle axis, so a novel feed system with additional rotating feed axis is developed to solve this issue. This section is divided into two parts which describes the concrete structure and the computation of the radial thermal error components respectively.

### The concrete structure of the feed system

**Fig. 3:**
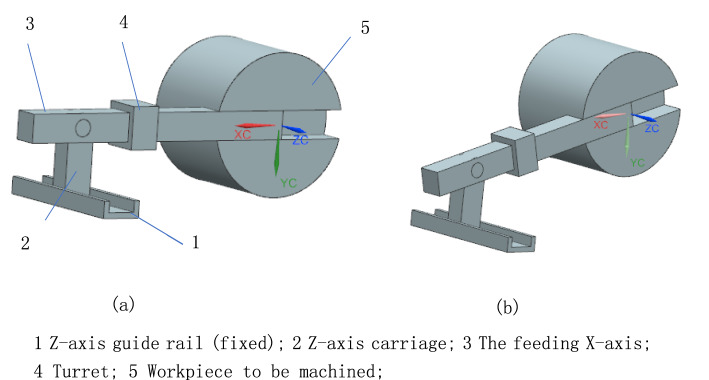
The kinematic diagram of mechanism for the 3-axis feed system (Created using NX12.0, it is available at the URL: https://plm.sw.siemens.com/zh-CN/nx/).

The root cause that the radial thermal error cannot be perfectly compensated is that the feeding X-axis does not intersect with the spindle axis, which is caused by thermal deformation of the machine tool structure. To address this, a rotating feed axis is added to the traditional feeding system, enabling it to rotate the X-axis dynamically and continuously to intersect with the spindle axis during thermal deformation. The working principle of the new feeding system can be illustrated by its kinematic diagram of mechanism as shown in Fig. 3. Z-axis guide rail (labeled 1) is fixed on the lathe bed, and the movement of workpiece (labeled 5) represents the thermal movement of spindle axis. The feeding X-axis (labeled 3) can rotate about rotational feed axis on the Z-axis carriage (labeled 2). No matter how the feeding X-axis rotates, the distance of turret (labeled 4) from the origin of the feeding X-axis remains unchanged. Figure 3a and b show the states without and with thermal movement of the spindle axis respectively. The feeding X-axis remains through the spindle axis wherever the spindle axis moves. The rotational feed axis is parallel to the feeding Z-axis and perpendicular to the feeding X-axis. Consequently, during the compensation of the radial thermal error, the position of the cutting tool along the feeding Z-axis remains unaffected, and the original accuracy on the Z-axis is preserved after compensation.

**Fig. 4:**
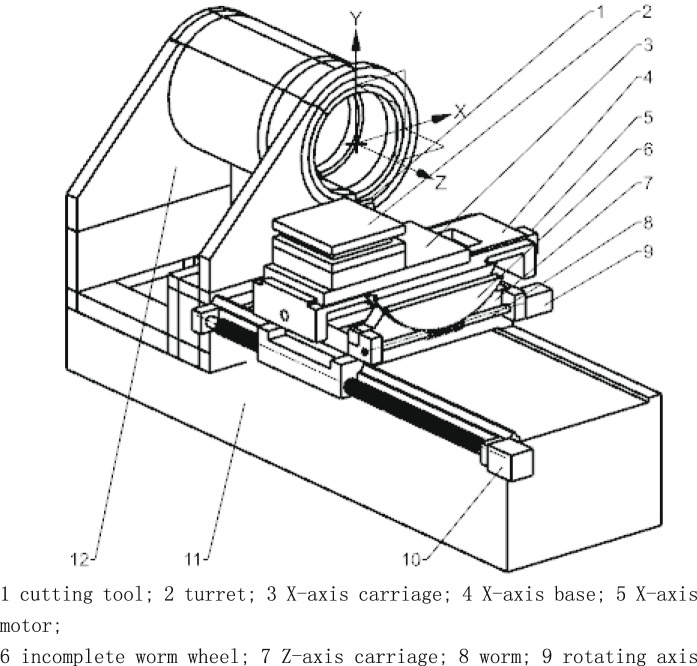
The schematic of a turning center with a three-axis feed system.

According to Fig. 3, a concrete physical structure for the 3-axis feed system can be designed. Different from the kinematic diagram of mechanism, for the factual 3-axis feeding system, the feeding X-axis remains through the spindle axis by driving the rotational feed axis at an angle that can be computed using the thermal error models as described in Section "Compensation for the radial thermal error". Considering the higher transmission ratio and space limitations, the worm and incomplete worm wheel mechanism is used to drive the rotating feed axis. The schematic assembly drawing of a turning center equipped with this feed system is provided as Fig. 4.

The whole feed system comprises a cutting tool turret and three feed axis components: the X-axis component, the rotating axis component, and the Z-axis component. The X-axis component includes the X-axis carriage (labeled 3 in Fig. 4), X-axis base (labeled 4), and X-axis motor (labeled 5). The rotating axis component, consisting of the X-axis base (reused from the X-axis component), an incomplete worm wheel (labeled 6), Z-axis carriage (labeled 7), worm (labeled 8), and rotating axis motor (labeled 9), is designed to rotate around a fixed center. The Z-axis component includes the Z-axis carriage, Z-axis motor (labeled 10), and lathe bed (labeled 11).

On the top side of the X-axis base, there is an X-axis guideway that allows the X-axis carriage to move with the actions of the X-axis motor, realizing the X-axis feed movement. Below the X-axis base, a convex circular guideway matches a concave circular guideway on the top side of the Z-axis carriage. The rotating axis motor, assembled with the Z-axis carriage, drives the X-axis base to revolve around the center of the circular guideway via the incomplete worm wheel and worm mechanism. Notably, the center of this circular guideway lies on the X-axis, meaning that this revolution does not alter the cutting tool’s position on the X-axis, simplifying subsequent computations for thermal error compensation.

On the top side of the lathe bed, a Z-axis guideway guides the Z-axis carriage, which is driven by the Z-axis motor to realize the Z-axis feed movement of the cutting tool.

### The computation of the radial thermal error components

The feeding movements of the feeding axes determine both the positions of the cutting tool and, consequently, the dimensions of the machined workpiece. Similarly, the thermal error should be decomposed into components related to these feeding axes so that it can be compensated through the coordinate feeding movements of the related axes. Typically, the number of thermal error components matches the number of feed axes in a machine tool. For a specific thermal error, the number of its components depends solely on the number of feed axes that can compensate for it through their movements.

**Fig. 5:**
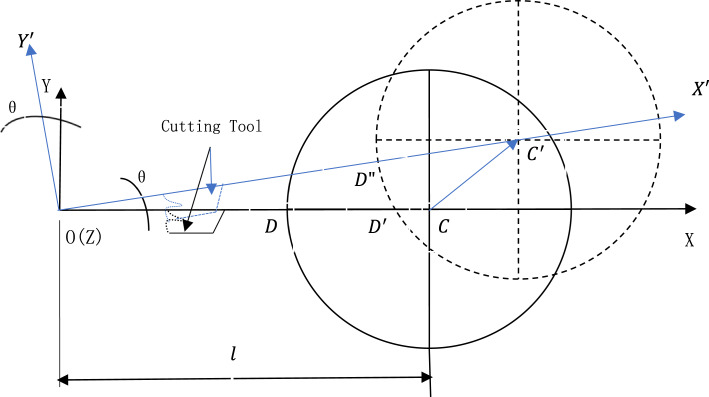
The computation for the radial thermal error components.

Under the assumption that the spindle axis takes translational movement during the thermal deformation, the radial thermal error components can be clearly found from Fig. 5 where the solid circle and the dotted circle represent the positions of the turned workpiece before and after thermal deformation respectively. The coordinate system and cutting tools in black and in blue represent the status before and after rotation of the rotating feed axis respectively. The origin of the coordinate system is the center of rotation of the rotating feed axis. The feeding X axis is designed to pass through the center of rotation of the rotating feed axis so that the radial thermal error component computation can be simplified.

For a turning center with the traditional two-axis feed system, the radial dimension of the workpiece is only determined by the X-axis feeding movement, so there is only one thermal error component for the radial thermal error ${E}_{r}$, that is, the X-axis feed movement from point $D$ to point ${D}^{\prime}$ that can be expressed as ${X}_{rad}$, and from Fig. 5 have:

8 $${E}_{r}={X}_{rad}=D{D}^{\prime}={C}_{x}^{\prime}-{C}_{x}+r-\sqrt{{r}^{2}-{{C}_{y}^{\prime}}^{2}}$$

where the subscripts $x$, $y$ denote the $x$ coordinate and $y$ coordinate of circle centers ${C}^{\prime}$ and $C$ respectively in the black coordinate system, $r$ is the radius of the machined workpiece. While for a turning center equipped with the proposed three-axis feed system, in order to assure the normal cutting conditions where the cutting tool tip passes through the spindle axis with the feeding movement of X-axis, there must be two thermal error components of which one is the linear component ${X}_{rad}$ (the X-axis feed movement) and the other is angular component ${R}_{rad}$ (the feed movement of the rotating feed axis). The radial thermal error ${E}_{r}$ can be expressed in the following form as a complex number:

9 $${E}_{r}={X}_{rad}{e}^{j{R}_{rad}}$$

It can be seen from Fig. 5, the rotational feed does not change the positions of the cutting tool on the X axis, and therefore it has:

10 $$\begin{aligned} X_{rad} = OC^{\prime} - OC &= \sqrt {\left( {l + \Delta x} \right)^{2} + \left( {\Delta y} \right)^{2} } - l \\ & \Delta x = C^{\prime}_{x} - C_{x} \\ & \Delta y = C^{\prime}_{y} - C_{y} \\ \end{aligned}$$

11 $${R}_{rad}=\theta ={\text{tan}}^{-1}\frac{\Delta y}{l+\Delta x}$$

where $$\Delta x$$ and $$\Delta y$$ are the X component and Y component of the thermal drift vector $$\overset{\lower0.5em\hbox{$\smash{\scriptscriptstyle\rightharpoonup}$}}{{CC^{\prime}}}$$ of the spindle axis respectively. $${C}_{y}^{\prime}$$ and $${C}_{y}$$ are the Y coordinates of center points $${C}^{\prime}$$ and $$C$$ respectively and here $${C}_{y}$$ is 0 since the X axis goes through the spindle axis before thermal deformation. $$\theta$$ is the rotational angle of the coordinate system around Z axis so that the after-rotated X axis ($$O{X}^{\prime}$$) can accurately go through the spindle axis. $$l$$ is the distance from the rotational center of the rotating feed axis to the spindle axis before thermal deformation. $$\theta$$ can be adjusted by change of $$l$$. $$\Delta x$$ and $$\Delta y$$ can be got by simulation or experimental method.

## Demonstration and analysis of compensation process

Simulation has been an important substitute for the experimental method in the field of thermally induced errors studies of machine tools due to its cost-effective and sufficiently reliable characteristics. In this section, the compensation process, demonstrated and analyzed through related data obtained by simulation, is presented on a turning center equipped with a three-axis feed system.

### Simulation of the thermal deformation of the headstock

**Fig. 6:**
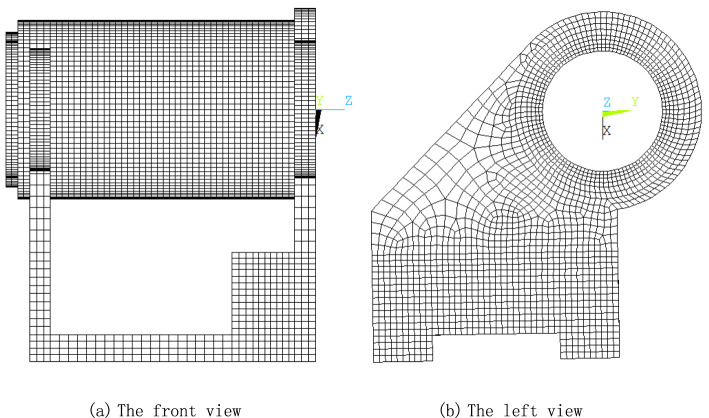
The finite element model for the headstock of a turning center.

For a turning center, due to lower feed velocity and better lubrication, the feed system structure itself typically results in fewer thermal errors. Therefore, the majority of thermal errors are caused by the deformation of the headstock, which results in the movement of the spindle axis. Consequently, only the thermal deformation process of the headstock is simulated. Figure 6a and b show the front and left views of the finite element model for the turning center headstock, respectively, with detailed mesh characteristics highlighted. The solid226 element from the elementary library of Ansys software is used. The primary heat sources are the rotations of the front and rear bearings. According to^18^, the heat generated is 102 W for the front bearing and 85.4 W for the rear bearing under the conditions of air cutting and a spindle rotating speed of 2000 rpm. The coefficient of convection heat transfer is assumed to be 10 W/(m^2^ K) based on practical experiences. Material properties include: density of 7.8 × 10^3^ kg/m^3^, Poisson’s ratio of 0.3, thermal capacity of 502.4 J/(kg K), thermal conductivity of 46.4 W/(m K), thermal expansion coefficient of 1.06 × 10^−5^ (1/K), and modulus of elasticity of 209 × 10^9^ N/m^2^. The machine tool’s work cycle is as follows: starting the machine tool to run for 3000 s, stopping for 1000 s, restarting to run for 4000 s, stopping again for 1000 s, and repeating until thermal balance is reached.

**Fig. 7:**
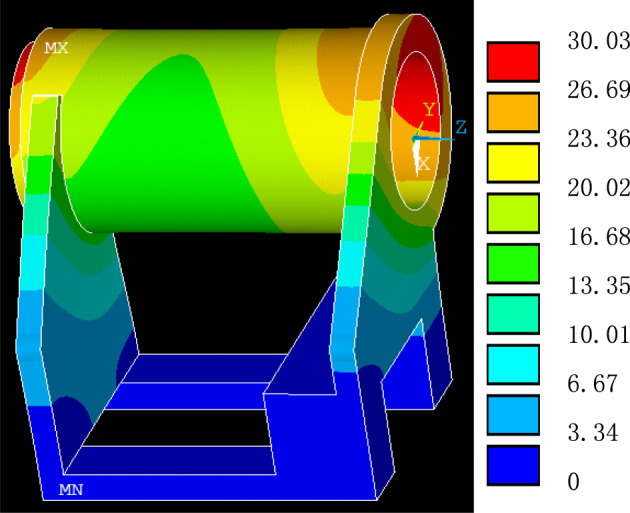
The temperature distribution of the headstock (Created using Ansys 2022 R2, it is available at the URL: https://www.ansys.com/academic.)

In this section, the transient thermal-structural coupling analysis provides critical insights into the temperature distribution and thermal deformation of the headstock throughout the machining process. In the thermal balance state illustrated in Fig. 7, the temperature peaks at the upper part of the spindle hole, decreasing towards the base. This indicates that air conducts heat less efficiently than the headstock material. An approximately circular temperature distribution around the spindle axis is got, which is advantageous for maintaining the circular integrity of the deformed spindle hole. Interestingly, the temperature rise at the headstock base is negligible, suggesting that it is reasonable to simulate just the headstock, omitting the lathe bed for data collection, despite the headstock’s direct contact with it.

**Fig. 8:**
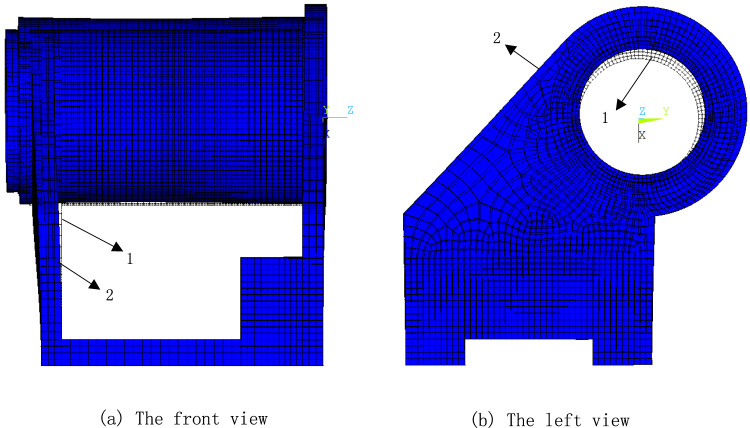
The thermal deformation of the headstock.

Figure 8 presents a comparison between the deformed and undeformed shapes of the headstock. The Original model (labeled 1), presented on a white background, is contrasted with the Deformed model (labeled 2), which is displayed on a blue background. As seen in Fig. 8a, the support rib plates on either side exhibit outward bending due to the thermal expansion of the spindle section between them. The front support rib plate experiences less bending than the rear, owing to its more robust structure, which effectively mitigates thermal errors along the spindle axis. Figure 8b highlights the displacement of the spindle hole, further emphasizing the impact of thermal effects on the headstock’s geometry.

### Movement analysis of the spindle axis

**Fig. 9:**
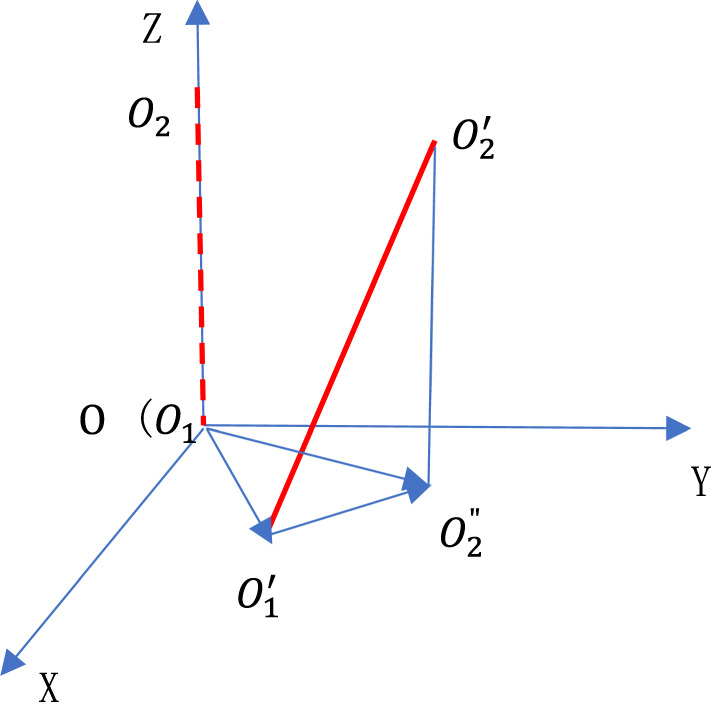
Movement of the spindle axis.

**Fig. 10:**
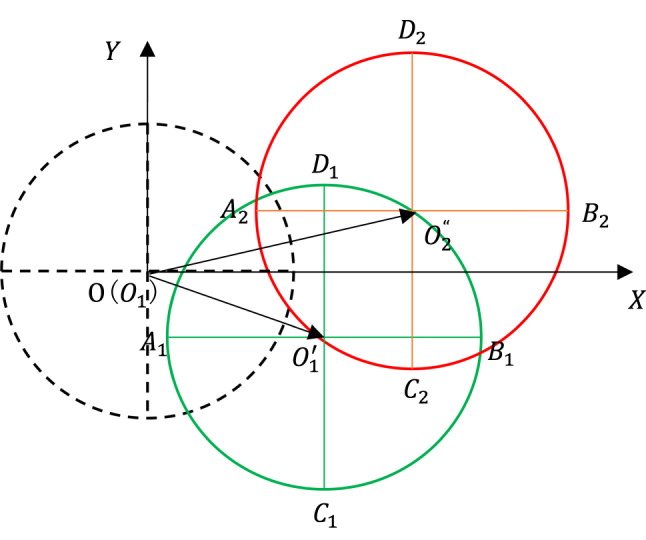
The computation of the vector $$\overset{\lower0.5em\hbox{$\smash{\scriptscriptstyle\rightharpoonup}$}}{{OO^{\prime}_{1} }}$$ and $$\overset{\lower0.5em\hbox{$\smash{\scriptscriptstyle\rightharpoonup}$}}{{OO^{\prime\prime}_{2} }}$$.

The spindle axis is defined as the line connecting the centers of the two circles at the end faces of the spindle hole. Figure 9, a skeletal drawing, illustrates the movement of the spindle axis. In this figure, the original spindle axis is represented by a red dashed line, with O₁ and O₂ denoting the centers of the circles at the two end faces before thermal deformation. The red solid line, on the other hand, depicts the spindle axis after thermal deformation, with $O^{\prime}_{1}$ and $O^{\prime}_{2}$ marking the new centers of the circles. Here, $O^{\prime\prime}_{2}$ signifies the projection of $O^{\prime}_{2}$ onto the XY coordinate plane where $O^{\prime}_{1}$ lies. Consequently, the deviation angle of the spindle axis from its original position can be expressed as the angle $\angle O^{\prime}_{1} O^{\prime}_{2} O^{\prime\prime}_{2}$:

12 $$\angle O^{\prime}_{1} O^{\prime}_{2} O^{\prime\prime}_{2} = \sin^{ - 1} \frac{{\left| {\overline{{O^{\prime}_{1} O^{\prime\prime}_{2} }} } \right|}}{{O^{\prime}O^{\prime}_{2} }}$$

where $${O}_{1}^{\prime}{O}_{2}^{\prime}$$ can take the length of the spindle axis because the axial thermal expansion is negligible compared with it, and the vector $$\overset{\lower0.5em\hbox{$\smash{\scriptscriptstyle\rightharpoonup}$}}{{O_{1}^{{\prime}} O_{2}^{\prime\prime} }}$$ is the projection of $${O}_{1}^{\prime}{O}_{2}^{\prime}$$ on the XY coordinate plane. From Fig. 10, have:

13 $$\overset{\lower0.5em\hbox{$\smash{\scriptscriptstyle\rightharpoonup}$}}{{O^{\prime}_{1} O^{\prime\prime}_{2} }} = \overset{\lower0.5em\hbox{$\smash{\scriptscriptstyle\rightharpoonup}$}}{{OO^{\prime\prime}_{2} }} - \overset{\lower0.5em\hbox{$\smash{\scriptscriptstyle\rightharpoonup}$}}{{OO^{\prime}_{1} }}$$

The vectors $$\overset{\lower0.5em\hbox{$\smash{\scriptscriptstyle\rightharpoonup}$}}{{OO_{2}^{\prime\prime} }}$$ and $$\overset{\lower0.5em\hbox{$\smash{\scriptscriptstyle\rightharpoonup}$}}{{OO_{1}^{{\prime}} }}$$ can be computed from Fig. 10. Before thermal deformation, the spindle axis is aligned with Z-axis, so the projections of the two end faces of the spindle hole on the XY coordinate plane coincide and are represented by a single dashed circle in Fig. 10. After thermal deformation, they are approximately represented by the green and red circles respectively. $${A}_{1}$$, $${B}_{1}$$, $${C}_{1}$$, $${D}_{1}$$, and $${O}_{1}^{\prime}$$ are the leftmost, rightmost, lowest, highest points and the center of the green circle respectively. Similarly, $${A}_{2}$$, $${B}_{2}$$, $${C}_{2}$$, $${D}_{2}$$, and $${O}_{2}^{\prime\prime}$$ are the leftmost, rightmost, lowest, highest points and the center of the red circle respectively. So the coordinate ($${O}_{1\text{x}}^{\prime}$$, $${O}_{1\text{y}}^{\prime}$$) of the circle center $${O}_{1}^{\prime}$$ on the XY coordinate plane can be expressed as:

14 $${O}_{1\text{x}}^{\prime}={A}_{1}\left(x\right)+\frac{{B}_{1}\left(x\right)-{A}_{1}\left(x\right)}{2}$$

15 $${O}_{1\text{y}}^{\prime}={C}_{1}\left(y\right)+\frac{{D}_{1}\left(y\right)-{C}_{1}\left(y\right)}{2}$$

where ${A}_{1}\left(x\right)$, ${B}_{1}\left(x\right)$ represent the X coordinates of ${A}_{1}$, ${B}_{1}$ respectively, and ${C}_{1}\left(y\right)$, ${D}_{1}\left(y\right)$ represent the Y coordinates of ${C}_{1}$, ${D}_{1}$ respectively. Similarly, the coordinate (${O}_{2x}^{\prime\prime}$, ${O}_{2\text{y}}^{\prime\prime}$) of the circle center ${O}_{2}^{\prime\prime}$ on the XY coordinate plane can be expressed:

16 $${O}_{2x}^{\prime\prime}={A}_{2}\left(x\right)+\frac{{B}_{2}\left(x\right)-{A}_{2}\left(x\right)}{2}$$

17 $${O}_{2\text{y}}^{\prime\prime}={C}_{2}\left(y\right)+\frac{{D}_{2}\left(y\right)-{C}_{2}\left(y\right)}{2}$$

where $${A}_{2}\left(x\right)$$, $${B}_{2}\left(x\right)$$ represent the X coordinates of $${A}_{2}$$, $${B}_{2}$$ respectively, and $${C}_{2}\left(y\right)$$, $${D}_{2}\left(y\right)$$ represent the Y coordinates of $${C}_{2}$$, $${D}_{2}$$ respectively. So the vector $$\overset{\lower0.5em\hbox{$\smash{\scriptscriptstyle\rightharpoonup}$}}{{OO^{\prime}_{1} }}$$ and $$\overset{\lower0.5em\hbox{$\smash{\scriptscriptstyle\rightharpoonup}$}}{{OO^{\prime\prime}_{2} }}$$ can be expressed by the following:

18 $$\overset{\lower0.5em\hbox{$\smash{\scriptscriptstyle\rightharpoonup}$}}{{OO^{\prime}_{1} }} =\left[\frac{{B}_{1}\left(x\right)+{A}_{1}\left(x\right)}{2}, \frac{{D}_{1}\left(y\right)+{C}_{1}\left(y\right)}{2} \right]$$

19 $$\overset{\lower0.5em\hbox{$\smash{\scriptscriptstyle\rightharpoonup}$}}{{OO^{\prime\prime}_{2} }} =\left[\frac{{B}_{2}\left(x\right)+{A}_{2}\left(x\right)}{2}, \frac{{D}_{2}\left(y\right)+{C}_{2}\left(y\right)}{2}\right]$$

${A}_{1}\left(x\right)$, ${B}_{1}\left(x\right)$, ${C}_{1}\left(y\right)$, ${D}_{1}\left(y\right)$, ${A}_{2}\left(x\right)$, ${B}_{2}\left(x\right)$, ${C}_{2}\left(y\right)$, ${D}_{2}\left(y\right)$ can be gotten by the simulation or experimental method, and in this paper they all come from the simulation computation. Combining the formula from (12) to (19), the deviation angle of the spindle axis can be solved. The computation results show the biggest deviation angle is 0.0099 degree, so in most cases it is feasible to regard the movement of the spindle axis as translational movement, and therefore either the group of ${A}_{1}\left(x\right)$, ${B}_{1}\left(x\right)$, ${C}_{1}\left(y\right)$, ${D}_{1}\left(y\right)$ or the group of ${A}_{2}\left(x\right)$, ${B}_{2}\left(x\right)$, ${C}_{2}\left(y\right)$, ${D}_{2}\left(y\right)$ can be selected to independently determine the position of the spindle axis.

### Compensation for the radial thermal error

Here the main works for compensating the radial thermal error involve computation of the real-time positions of the spindle axis; determining the two radial thermal error components that correspond to each position of the spindle axis; modeling the thermal error for these two components separately; and analyzing prediction accuracy of thermal error components. Given the assumed translational movement of the spindle axis, the positions of the spindle axis can be determined by use of either the positions of ${A}_{1}\left(x\right)$, ${B}_{1}\left(x\right)$, ${C}_{1}\left(y\right)$, ${D}_{1}\left(y\right)$ or the positions of ${A}_{2}\left(x\right)$, ${B}_{2}\left(x\right)$, ${C}_{2}\left(y\right)$, ${D}_{2}\left(y\right)$. Comparing the formula (10) to (11) with formula (14) to (15), or alternatively with formula (16) to (17), it can be known that $\Delta x$ and $\Delta y$ in the formula (10) to (11) represent ${O}_{1\text{x}}^{\prime}$ and ${O}_{1\text{y}}^{\prime}$ in the formula (14) to (15) respectively, or ${O}_{2x}^{\prime\prime}$ and ${O}_{2\text{y}}^{\prime\prime}$ in the formula (16) to (17), so the real-time practical linear components ${X}_{rad}$ and angular components ${R}_{rad}$ of the radial thermal error ${E}_{r}$ can be computed according to the positions of the spindle axis. The conventional multivariate regression analysis method is used to build the prediction models for the two radial thermal error components, with the temperatures at two measuring point, ${T}_{1}$ and ${T}_{2}$, as variables. The two prediction models can be expressed as follows:

20 $$P{X}_{rad}={C}_{1}{T}_{1}+{C}_{2}{T}_{2}+C$$

21 $$P{R}_{rad}={D}_{1}{T}_{1}+{D}_{2}{T}_{2}+D$$

where $P{X}_{rad}$ and $P{R}_{rad}$ are the predicted values for ${X}_{rad}$ and ${R}_{rad}$ respectively. ${C}_{1}$, ${C}_{2}$, $C$, ${D}_{1}$, ${D}_{2}$ and $D$ are to-be-solved coefficients that can be solved using least square method in Matlab software. In order to demonstrate the effects of *l* on the two radial thermal error components, the studies are carried out under the different value of* l*, and correspondingly the two radial thermal error components models can be got respectively as follow:

For $$l = 0.2\;{\text{mm}}$$, have

$$\begin{aligned} & PX_{rad} = 0.0004T_{1} + 0.002T_{2} - 0.0018 \\ & PR_{rad} = 0.3014T_{1} + 0.3238T_{2} + 0.526 \\ \end{aligned}$$

For $$l = 0.5\;{\text{mm}}$$, have

$$\begin{aligned} & PX_{rad} = 0.0003T_{1} + 0.002T_{2} - 0.0009 \\ & PR_{rad} = 0.1305T_{1} + 0.1520T_{2} + 0.0796 \\ \end{aligned}$$

For $$l = 1\;{\text{mm}}$$, have

$$\begin{aligned} & PX_{rad} = 0.0002T_{1} + 0.0019T_{2} - 0.0006 \\ & PR_{rad} = 0.0669T_{1} + 0.08T_{2} + 0.0173 \\ \end{aligned}$$

**Fig. 11:**
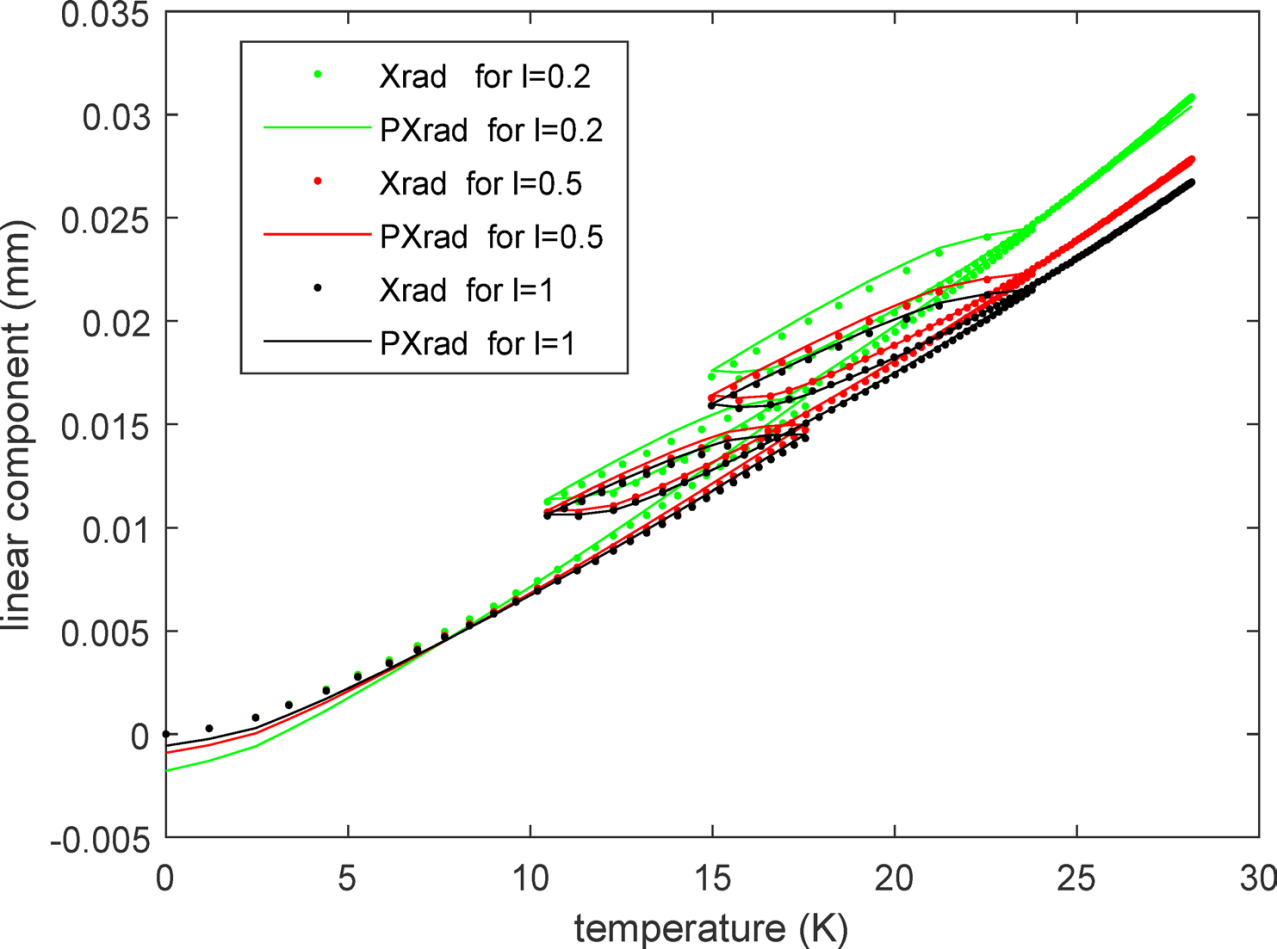
The fitting curves for the radial linear thermal error component.

**Fig. 12:**
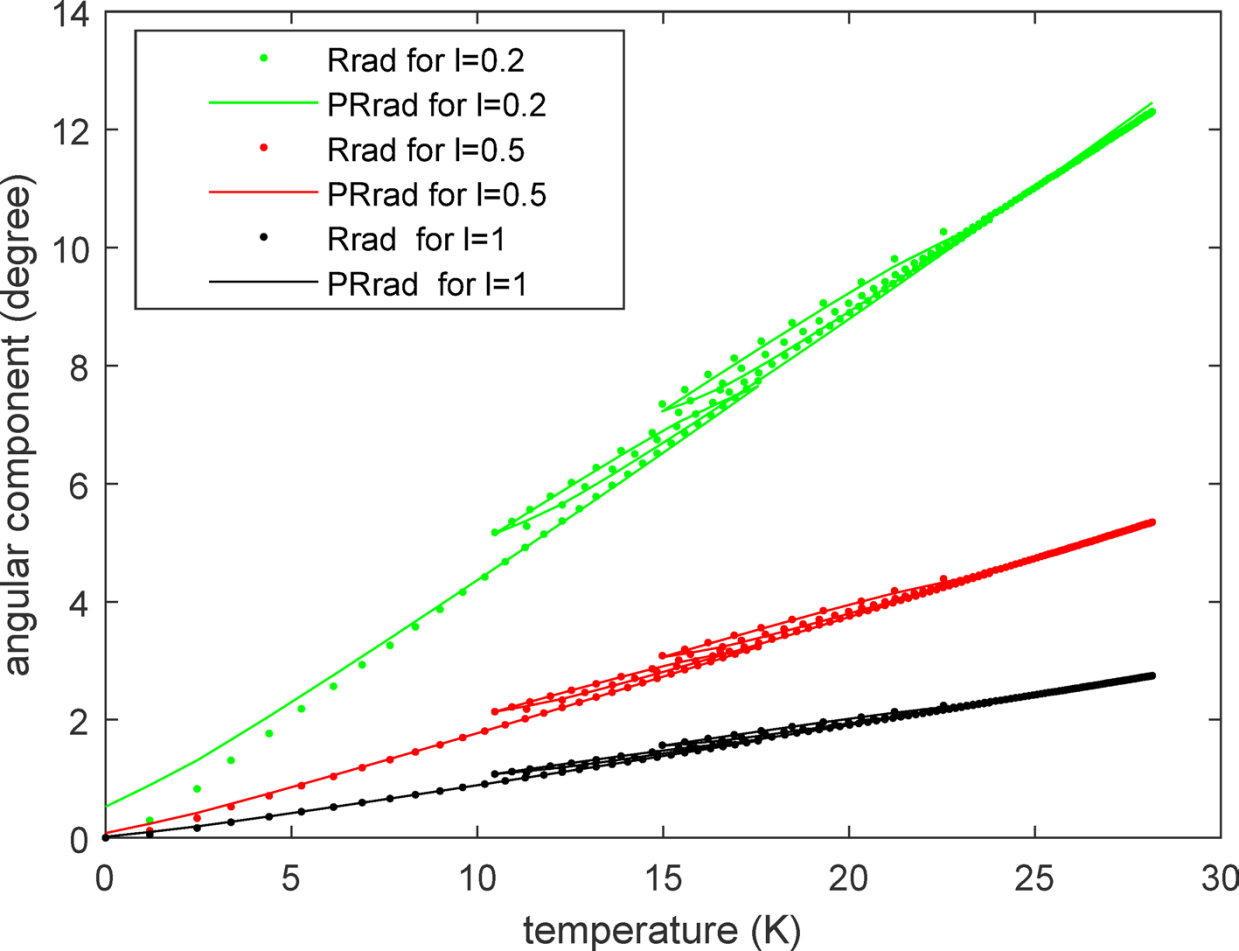
The fitting curves for the radial angular thermal error component.

The fitting curves for the linear component and angular component of the radial thermal error are displayed in Figs. 11 and 12 respectively. It can be observed that the predicted values correspond well with the practical values for both the linear and angular components across three different values of '*l*'. Notably, small changes in '*l*' result in larger variations in the angular component compared to the linear component. This is advantageous for enhancing the flexibility of application of the three-axis feed system, as the angular scope is often constrained by practical considerations such as limited space or motion accuracy. As in formula (9), the predicted radial thermal error ${PE}_{r}$ for ${E}_{r}$ can also be written as the form of a complex number:

22 $${PE}_{r}={PX}_{rad}{e}^{j{PR}_{rad}}$$

**Fig. 13:**
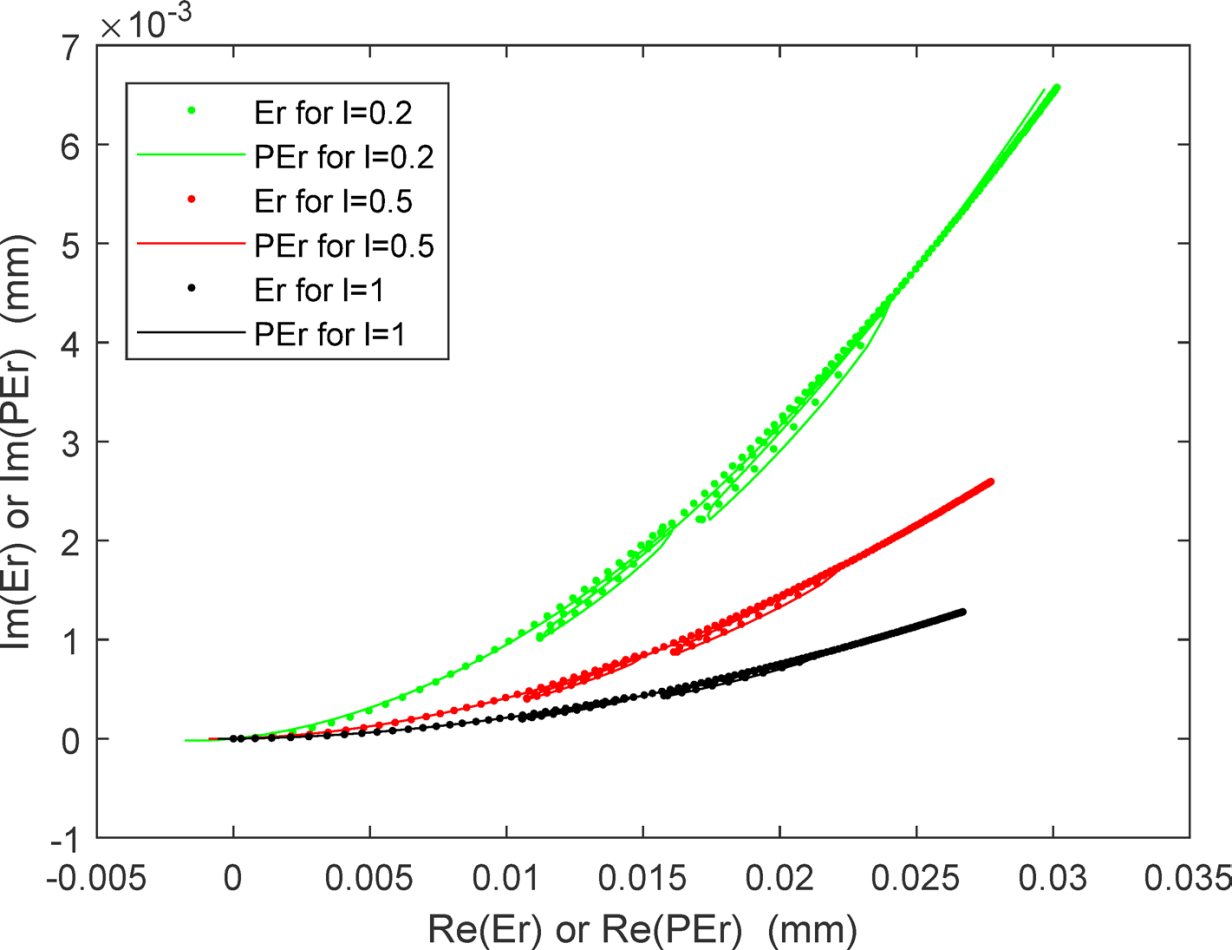
The motion route of the radial thermal error ${PE}_{r}$ and ${E}_{r}$.

The traces of both ${PE}_{r}$ and ${E}_{r}$ are displayed in Fig. 13 where the horizontal and vertical axes represent the real part and imaginary part, respectively, of the complex number. Therefore, the position of each point on these traces conveys information of the linear and angular components of the radial thermal error. The changes in the linear component relative to the angular component of the radial thermal error can be clearly observed from Fig. 13 alone. The smaller the value of *l* is, the larger the revolving angle becomes, which translates to greater space requirements. Conversely, a larger value of *l* results in a smaller revolving angle, which can lead to issues of poor resolution in the driving system, especially when the actual angle itself is small due to the relatively modest magnitude of thermal deformation. Therefore, caution should be exercised when determining the value of *l*.

## Conclusions

This paper presents a three-axis feed system of a turning center that can be used to control the radial error, of machined parts, caused by the thermal deformation of the turning center headstock. The following specific conclusions can be drawn:

1. The radial thermal error of the turning center cannot be fully compensated for by a feed system with only two feed axes;
2. Due to the presence of a revolving feed axis in the three-axis feed system, the cutting tool’s tip can be maintained to pass through the spindle axis as it feeds along the X axis through the interpolation movement of the X axis and the revolving axis. This allows for improved surface finish on machined parts with larger radial dimensions and avoids compensation failures for extremely small radial dimensions when using this feed system;
3. The evaluation method for the spindle axis movement is provided, and the results show that the spindle axis movement can be regarded as translational movement.
4. The computational methods for the linear and angular components of the radial thermal error components are provided based on the movement behavior of the three-axis feed system. Compensation results show that the impact of parameter *l* on the angular component is greater than its impact on the radial component. Adjusting parameter *l* can enhance the feed system’s adaptability to various in-situ conditions.
5. The three-axis feed system will turn into a traditional two-axis feed system when locking the revolving feed axis. This feature allows it to conveniently replace existing two-axis feed systems, as aligning the X-axis with the spindle axis becomes straightforward, requiring only a single movement of the revolving feed axis.

## Data Availability

The data used and/or analysed during the current study comes from experimental measuring and simulation, which can be available from the corresponding author on reasonable request.
